# When the FAT goes wide: Right extended Frontal Aslant Tract volume predicts performance on working memory tasks in healthy humans

**DOI:** 10.1371/journal.pone.0200786

**Published:** 2018-08-01

**Authors:** Federico Varriano, Saül Pascual-Diaz, Alberto Prats-Galino

**Affiliations:** Laboratory of Surgical Neuroanatomy, University of Barcelona, Barcelona, Spain; Chinese Academy of Sciences, CHINA

## Abstract

The Frontal Aslant Tract (FAT) is a tract recently described as having implications on language function. The originally proposed anatomical FAT definition characterizes a connection between Broca’s territory and anterior supplementary and pre-supplementary motor areas in the Superior Frontal Gyrus (SFG). Here we propose an extended definition of the FAT (the exFAT) that propagates more anteriorly into the SFG. A sample of 834 subjects from the WU-Minn HCP 900 subjects data release (S900) was selected. The bilateral exFATs were reconstructed for the whole sample using an automated pipeline and thresholded adjusted tract volumes were calculated. A laterality test was performed on the whole sample. The frontal cortex has known implications on superior cognitive functions, so here we evaluate the implications of exFAT volume on performance in a language task and on a set of working memory tasks. Two sub-samples of 70 subjects each were drawn from the S900 sample by selecting the 35 top-performers and 35 bottom-performers for both language and working memory tasks. Additional laterality tests were performed on each subsample. We did not find the exFAT to be lateralized in any of the samples. We found statistically significant differences in left adjusted exFAT volume between top-performers and bottom-performers in the language task. We also found statistically significant differences in right adjusted exFAT volume between top-performers and bottom-performers for 2-back working memory tasks. To check for the predictive power of the exFAT volumes as correlates for performance, we ran a repeated random sub-sampling cross-validation procedure based on a Support Vector Machine (SVM) classifier that was capable of correctly classifying holdout subjects to their corresponding group (top-performer vs bottom-performer) with an average accuracy of 74.5% for language task performance based on left exFAT volume and an accuracy of 64.2% for Working Memory performance based on right exFAT volume.

## Introduction

The Frontal Aslant Tract (FAT) is a novel brain tract first described by Catani *et al*. in virtual dissection studies validated against blunt dissection [1]. The FAT is described as a left-lateralized bilateral tract connecting Broca’s territory (specifically *Pars Opercularis* (POp), with some connections reaching *Pars Triangularis* (PTr)) and the supplementary and pre-supplementary motor areas of the Superior Frontal Gyrus (SFG). A homologous tract has been described in monkeys [2] and its existence has been further corroborated in dissection and tractographic studies [3].

Given that the FAT connects Broca’s territory with motor regions in the left hemisphere, it has been posited that the left FAT could have functional implications in motor aspects of language. Numerous studies yield results confirming this hypothesis: The left FAT integrity has been shown to be reduced in Primary Progressive Aphasia patients [4] and its damage predicted speech fluency alterations in chronic post-stroke aphasia [5]. Additionally, intraoperative electrical stimulation of the left FAT territory in awake brain surgery induces transient stuttering [6] and speech arrest [7,8]. Patients who underwent surgical resection of tumors in the left FAT territory presented speech and motor initiation disorders shortly after the surgery. These disorders spontaneously reverted after 3 months, with most patients recovering their baseline speech and motor functions [6,9]. Some evidence points to additional cognitive implications of the FAT: Sierpowska *et al*. showed that the left FAT could be involved in the application of morphological derivation rules in speech production [10].

Evidence elucidating the functional role of the right FAT is sparser: It has been shown that it could be involved in voluntary hand movement [11] and its lesion could induce transient motor inhibition [9], but overall its function is not yet well understood.

The late discovery of the FAT compared to classically described neural pathways is in part explained by the deceivingly non-eloquent nature of the frontal lobe, once labeled the “silent lobe”, as Bozkurt *et al*. reports [12]. Furthermore, the complex white matter architecture of the FAT territory makes its tractographic reconstruction challenging under traditional tensor-based tractographic techniques. Thus, two critical conditions for proper tractographic FAT assessment become apparent: a) advanced diffusion models should be used to minimize artefactual reconstructions and false negatives, and b) a large sample size becomes paramount to achieve enough statistical power as to accurately assess relationships between FAT’s anatomy and its function.

One central aspect of tractography reconstruction is the selection of ROIs (regions of interest), as it often involves manual procedures which can be time-consuming and require expert anatomist knowledge as well as special care to control for operator-induced variability. The originally proposed methodology for FAT reconstruction involves the manual placement of ROIs, making the analysis of large samples unfeasible.

Regarding ROI placement for FAT delineation, a less restrictive criteria consisting of “an axial AND ROI around the white matter of the SFG and a sagittal AND ROI around the white matter of the IFG including pars opercularis, triangularis and orbitalis” has been described [11]. Nonetheless, the reported FAT reconstructions do not show a trajectory reaching more anterior regions, making us think that the SFG white matter ROI was selected around supplementary and pre-supplementary motor areas and not around the full extension of the white matter of the SFG.

### Introducing the extended FAT (exFAT)

Here, we propose an extended FAT definition (the exFAT) composed of fibers explicitly connecting Broca’s territory (defined as POp and PTr) with the totality of the SFG, reaching anterior superior frontal regions which are known for having functional implications in high order cognitive functions such as working memory: Chronic spatial working memory deficits have been reported in patients after tumor resection in the right prefrontal parenchyma [13].

The rationale for defining the exFAT is twofold: first, it allows us to readily leverage the power of automated segmentation systems in a large high quality sample in a replicable way while avoiding operator-induced biases. Secondly, given that evidence shows that working memory systems and language function are mediated by related cognitive abilities [14–16], the exFAT could be a reasonable candidate for a tract subserving both functions.

To our knowledge there is no compelling evidence in the literature that the FAT alone is involved in working memory function. Although not directly related to the study of frontal aslant connections, studies looking for statistical relationships between left hemisphere white matter volume and language function, and right hemisphere white matter volume and high order cognitive functions have been conducted. For example, it has been shown that white matter volume of Brodmann Area 39 in the right hemisphere is associated with higher IQ [17], and it has been shown in a Voxel-Based Morphometry study that fast learners presented higher white matter density in left Heschl’s Gyrus than slow learners in a foreign speech phonetic learning task [18].

Given this extended definition, and according to the presented evidence, we hypothesize that a) the left exFAT mediates language performance, and b) the right exFAT mediates working memory performance.

## Material and methods

For the present study, a total of 834 healthy subjects were selected from the WU-Minn Human Connectome Project (HCP) 900 data release (S900) preprocessed dataset [19] after discarding those who lacked diffusion acquisitions or relevant tests scores. This study was conducted with the approval of the Bioethics Committee of the University of Barcelona. Institutional Review Board (IRB00003099).

We developed a fully automated pipeline based on the standard MRTrix3 [20] HCP pipeline that reconstructs the bilateral exFAT. Tissue-segmented images were generated for Anatomically Constrained Tractography. Multi-Shell Multi-Tissue Constrained Spherical Deconvolution was used in order to obtain the exFAT tractographic reconstructions.

The HCP dataset offers high quality atlases containing FreeSurfer [21] parcellations and segmentation maps that are aligned to the subjects diffusion space while being robust against potential systematic biases that FreeSurfer could show when parcellating individual subjects. For each hemisphere, atlas labels were used to define seed regions as the sum of POp and PTr. The SFG ROI was used as an inclusion mask and every other gray matter structure other than the two explicitly defined as seed and inclusion was employed as an exclusion mask to produce automated and robust tract reconstructions while reducing the presence of false positive tracts [22].

Tracts were reconstructed in subjects native space using the iFOD2 algorithm [23] with recommended MRTrix3 parameters for HCP datasets and 1M fibers per tract were fired. A maximum length limit of 150mm was defined to reduce the presence of false positives. Density maps were calculated from the exFAT tractographies and a hard threshold of 10 fibers was applied using FSL [24] to exclude artefactual false positive reconstructions that would bias the tract volume measure while preserving the main tract bundle. Left and right exFAT volumes were calculated from the thresholded density maps. Adjusted exFAT tract volume indices were calculated to control for sex induced volume differences by dividing the tract volume by the total brain volume; the resulting dimensionless quantities were multiplied by 10^6^ for scaling convenience.

For cognitive functions assessment, we used the average accuracies from the Binder *et al*. language processing task [25] for language assessment and both 0-back and 2-back working memory tasks for working memory assessment as available in the S900 sample. A summary of samples descriptors can be found in Table 1.

**Table 1:** Description of the samples.

Group	N	Age	Handedness	Language task score	Working memory 2-back task score
**Global sample**	834	28.78(3.86)	65.43(44.69)	88.78(6.97)	83.44(10.80)
**Language bottom performers**	35	28.46(3.85)	66.43(55.26)	71.3(4.38)	75.45(11.97)
**Language top performers**	35	28.29(2.83)	59.71(39.60)	100(0)	90.48(5.79)
**Working memory bottom performers**	35	30.11(3.81)	75.43(44.54)	81.50(14.32)	55.98(5.93)
**Working memory top performers**	35	27.89(3.69)	80.00(15.31)	93.54(4.19)	98.22(0.94)

Language task scores and working memory 2-back task scores are shown in a 0–100 scale. Standard deviations are shown in parentheses.

A laterality test was performed on the global sample using Welch’s t-test to compare left and right adjusted exFAT volume indices while correcting for variances inequality.

To study the exFAT implication in language function and working memory, two extreme subsamples were created:

1. Language extreme group: A sample of 70 subjects was constructed by selecting the 35 bottom-performers and the 35 top-performers on the adjusted language task test.
2. Working memory extreme group: A sample of 70 subjects was constructed by selecting the 35 bottom-performers and the 35 top-performers on the 2-back working memory test.

In the extreme groups, correlations were calculated for the relevant task score between top-performers and bottom-performers. Handedness and age were used as covariates for the language groups correlations and age alone was used as a covariate for the working memory groups correlations. Welch’s t-tests were applied to check for differences in average adjusted exFAT volume indices between top-performers and bottom-performers for left and right exFAT (performance test), and between left and right exFAT for top-performers and bottom-performers (laterality test).

Statistical tests were performed using R statistical software [26]. A Bonferroni-corrected significance threshold of α = 0.00156 was established for H_0_ rejection, corresponding to 32 comparisons at an original threshold of α = 0.05.

Finally, to further confirm the validity of the results, we ran a repeated random sub-sampling cross-validation procedure in Python [27] with a Support Vector Machine (SVM) model using SVC module from SKLearn library [28] with a linear kernel. A holdout ratio of 0.2 was used and 100 repetitions were considered for each result validation.

## Results

The adjusted volume indices for the global sample and extreme subsamples can be found at Table 2. Additional descriptors for characterization of the global sample can be found at S1 Fig.

**Table 2:** Adjusted volume indices for exFAT samples.

Group	N	Left adjusted exFAT volume indices	Right adjusted exFAT volume indices
**Global sample**	834	21.33(7.17)	20,90(6.34)
**Language bottom performers**	35	18,06(7,25)	18,46(7.08)
**Language top performers**	35	23.77(5.53)	23.15(5.29)
**Working memory bottom performers**	35	19.07(8.21)	17.76(7.52)
**Working memory top performers**	35	22.84(5.85)	23.4(6.0)

Adjusted volume indices were calculated by dividing tract volume by total brain volume and multiplying by 10^6^. Standard deviations are shown in parentheses.

### Global sample results

We did not find a significant difference in the adjusted volume indices between the left exFAT (M = 21.33, SD = 7.17) and right exFAT (M = 20.90, SD = 6.34) in the global sample; t(1641.7) = 1.304, p = 0.192. Data shown in Table 3.

**Table 3:** Lateralization test for global sample.

	Left		Right		t-test	
	M	SD	M	SD	t(df)	p
**Global sample**	21.33	7.17	20.90	6.34	1.304(1641.7)	0.192

M, group mean; SD, group standard deviation; t, value of Welch’s t statistic; df, estimated degrees of freedom; p, p-value.

**Fig 1:**
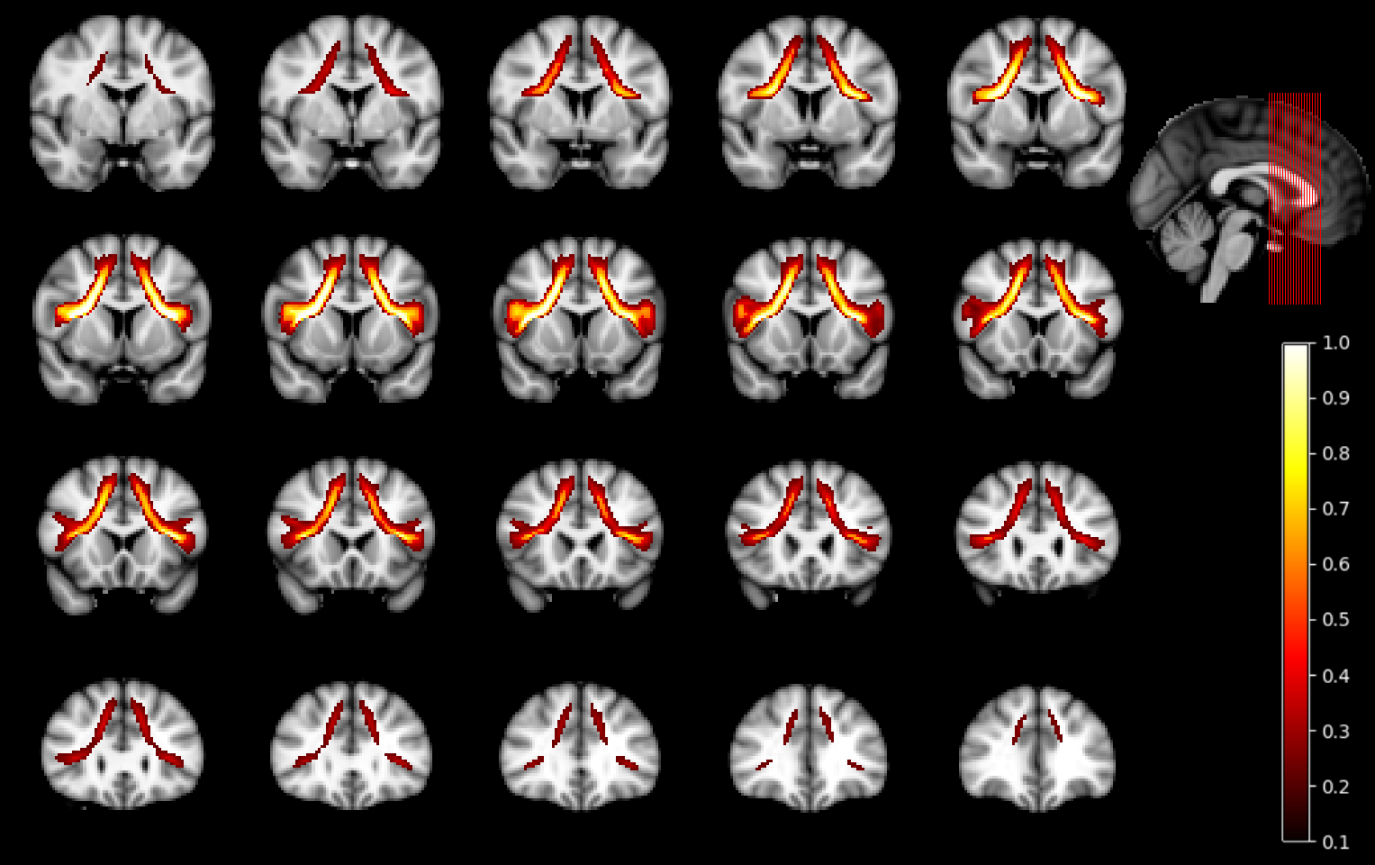
Bilateral exFAT probability map. Probability map showing average fiber density along the bilateral exFAT trajectory. Values are only shown in voxels present in >10% of subjects.

A probability map depiction of the exFAT trajectory is shown in Fig 1.

### Language extreme results

We found statistically significant correlations between language task performance and the bilateral adjusted exFAT volume indices. Data shown in Table 4.

**Table 4:** Language task vs adjusted volume index correlations.

	Left adjusted exFAT volume index	Right adjusted exFAT volume index
**Language task performance**	0.460*	0.444*

Age and handedness were used as covariates.

* indicates H_0_ rejection at p<0.0015625.

We did not find a significant difference in the adjusted volume indices between the left exFAT and right exFAT neither in the bottom-performers language task group (Mleft = 18.06, SDleft = 7.25; Mright = 18.46, SDright = 7.08) nor in the top-performers language task group (Mleft = 23.77, SDleft = 5.53; Mright = 23.15, SDright = 5.29). Data shown in Table 5.

**Table 5:** Lateralization tests for language samples.

	Left		Right		t-Test	
	M	SD	M	SD	t(df)	p
**Language top performers**	23.77	5.53	23.15	5.29	0.481(67.859)	0.63
**Language bottom performers**	18.06	7.25	18.46	7.08	-0.234(67.961)	0.815

M, group mean; SD, group standard deviation; t, value of Welch’s t statistic; df, estimated degrees of freedom; p, p-value.

For the extreme language task groups, we found that the top-performers had a greater adjusted volume index than bottom-performers for the left exFAT: bottom-performers (M = 18.06, SD = 7.25); top-performers (M = 23.77, SD = 5.53); t(63.579) = -3.7083, p<0.001. We did not find a significant difference in the adjusted volume indices between top-performers and bottom-performers in the language task groups for the right exFAT: bottom-performers (M = 18.46, SD = 7.08); top-performers (M = 23.15, SD = 5.29); t(62.938) = -3.143, p = 0.0025. Data shown in Table 6.

**Table 6:** Performance tests in language samples.

	Low		High		t-Test	
	M	SD	M	SD	t(df)	p
**Language left exFAT**	18,06	7.25	23.77	5.53	-3.708(63,579)	0.00044*
**Language right exFAT**	18.46	7.08	23.15	5.29	-3.143(62.938)	0.0025

M, group mean; SD, group standard deviation; t, value of Welch’s t statistic; df, estimated degrees of freedom; p, p-value.

* Indicates H_0_ rejection at p<0.00156.

An additional boxplot representation of the left adjusted exFAT volume indices distribution for the language extreme groups can be found at S2 Fig.

#### Machine learning model for language extreme groups

The averaged results for the holdout sample in the SVM classifier-based repeated random sub-sampling cross-validation with a holdout fraction of 0.2 after 100 random permutations were: accuracy: 0.745 ± 0.984; AUC: 0.755 ± 0.094 for language task score vs adjusted left exFAT volume index.

### Working memory results

We did not find any statistically significant correlation between the 0-back working memory task and adjusted exFAT volume indices. We found statistically significant correlations between adjusted right exFAT volume indices and global working memory task accuracy, global 2-back working memory task accuracy and tool 2-back working memory task accuracy. Data shown in Table 7.

**Table 7:** Working memory vs adjusted volume index correlations.

	Left adjusted exFAT volume index	Right adjusted exFAT volume index
Global accuracy	0.264	0.408*
Global 0-back accuracy	0.26	0.336
Global 2-back accuracy	0.226	0.39*
Body 0-back accuracy	0.165	0.152
Body 2-back accuracy	0.172	0.282
Face 0-back accuracy	0.245	0.298
Face 2-back accuracy	0.163	0.355
Place 0-back accuracy	0.176	0.203
Place 2-back accuracy	0.219	0.329
Tool 0-back accuracy	0.109	0.222
Tool 2-back accuracy	0.162	0.399*

Age was used as a covariate

* Indicates H_0_ rejection at p<0.0015625

We did not find a significant difference in the adjusted volume indices between the left exFAT and right exFAT neither in the bottom-performers working memory task group (Mleft = 19.07, SDleft = 8.21; Mright = 17.76, SDright = 7.52) nor in the top-performers working memory task group (Mleft = 22.84, SDleft = 5.85; Mright = 23.4, SDright = 6). Data shown in Table 8.

**Table 8:** Lateralization tests for working memory samples.

	Left		Right		t-Test	
	M	SD	M	SD	t(df)	p
**Working memory top performers**	22.84	5.85	23.4	6.0	-0.417(67.956)	0.678
**Working memory** **bottom performers**	19.07	8.21	17.76	7.52	0.699(67.485)	0.487

M, group mean; SD, group standard deviation; t, value of Welch’s t statistic; df, estimated degrees of freedom; p, p-value.

For the extreme 2-back working memory task groups, we found that the top-performers had a greater adjusted volume index than the bottom-performers for the right exFAT: bottom-performers (M = 17.76, SD = 7.52) and top-performers (M = 23.4, SD = 6.0); t(64.791) = -3.487, p<0.001. We did not find a significant difference in the adjusted volume indices between top-performers and bottom-performers in the 2-back working memory task groups for the left exFAT: bottom-performers (M = 19.07, SD = 8.21); top-performers (M = 22.84, SD = 5.85); t(61.431) = -2.21; p = 0.031. Data shown in Table 9.

**Table 9:** Performance tests in working memory samples.

	Low		High		t-Test	
	M	SD	M	SD	t(df)	p
**Working memory** **left exFAT**	19.07	8.21	22.84	5.85	-2.21(61.431)	0.031
**Working memory right exFAT**	17.76	7.52	23.4	6.0	-3.487(64.791)	0.0009*

M, group mean; SD, group standard deviation; t, value of Welch’s t statistic; df, estimated degrees of freedom; p, p-value.

* Indicates H_0_ rejection at p<0.00156.

An additional boxplot representation of the right adjusted exFAT volume indices distribution for the working memory extreme groups can be found at S3 Fig.

#### Machine learning model for working memory extreme groups

The averaged results for the holdout sample in the SVM classifier-based repeated random sub-sampling cross-validation with a holdout fraction of 0.2 after 100 random permutations were: accuracy: 0.642 ± 0.128; AUC: 0.654 ± 0.123 for 2-back working memory task score vs right adjusted exFAT volume indices.

## Discussion

Although many advanced diffusion-based indices have been proposed to study microstructural integrity and functional implications of white matter connections, a clear consensus about how they characterize the FAT has not yet been reached. Evidence shows that in children the FAT shows little to no microstructural differences with age while the *Arcuate Fasciculus* does [29] and its characterization is still an open problem.

Given that the exFAT contains the original FAT and extends it, we employed tract volume as our reference index of anatomical variability given its methodological simplicity and robustness regarding to tractography reconstruction parameters. To solve the problem of multiple comparisons, we used the Bonferroni correction as we intended to apply the most conservative correction method that still detects a signal relating Language function with the left hemisphere aslant connections anatomy, which is a well-described relationship in the literature, and apply that rigorous criterion elsewhere in the study. It is true that a less stringent correction method could lead to a reduced risk of type II errors, but given the risk of inflating type I errors while reporting results that go beyond what can be found in the literature, we opted to err on the side of caution.

Controlling for total brain volume implicitly removes potential biases induced by sex. Given that the employed language task is designed to be easy and general [25] and that working memory training only produces short-term specific effects that do not generalize [30], education level was not included as a covariate: It should be expected that people with higher language capacity as measured by the Binder task and higher working memory capacity will reach, on average, a higher education level, while it is not clear that the opposite implication should be true. Then, covariating by education level could reduce the explanatory power of the model by subtracting a large proportion of the signal we intend to detect in the first place.

Further studies should be conducted to better characterize the microstructural properties of the frontal aslant connections using state-of-the-art diffusion indices.

### exFAT and language

Numerous studies on the FAT function have focused on the left FAT implications on language and motor function, which is a reasonable approach given the left-lateralization of language in most of the population. Our results suggest that the left exFAT mediates performance in language-related tasks. It must be noted that under the present approach, it is hard to determine which language functions the exFAT is actually subserving. Further studies should be conducted to better understand the implications of the left exFAT and its different parts in cognitive aspects of language function.

### exFAT and working memory

One of the main driving hypotheses behind the present study was that extending the anatomical definition of the FAT by allowing its course to take a more anterior trajectory into the SFG would allow it to reach high level cognitive areas such as the dorsolateral prefrontal cortex and thus making it sensitive to differences in domains such as working memory performance that are not detectable by measuring the FAT alone, while still being at least partially sensitive to differences attributed to the original FAT. Further studies should be conducted in order to determine whether the anterior part of the exFAT show the strongest implication in working memory performance.

Our results suggest that the right exFAT has implications in working memory: we have found that adjusted right exFAT volume index was significantly correlated with 2-back task accuracies, but not with 0-back task accuracies, and average right adjusted exFAT volume indices were higher for the top-performers than for the bottom-performers in the 2-back working memory task. This result is in line with Kinoshita *et al*., who have reported spatial working memory deficits in patients after right prefrontal glioma resection [13].

To our best knowledge, this represents a novel result regarding the functional implication of the frontal aslant connections that has not been previously reported by studying the FAT alone.

### Cross-validated ML model

For further validation of our results, we trained a SVM classifier in a repeated random sub-sampling cross-validation paradigm with a holdout fraction of 0.2 and 100 random permutations. The resulting accuracies indicate the ability to correctly classify the 20% subjects that have not been used for the algorithm training after using the remaining 80% as a training sample.

The accuracy results based on the SVM classifier model should to be taken in conjunction with the confirmatory data analyses provided. While a classification accuracy of 64.2% on its own is not suitable for a real life classification task, it should be emphasized that we were able to obtain an above-than-chance accuracy when averaging over a large number of permutations, and this increases our confidence that we are indeed measuring an actual underlying relationship between brain anatomy and function and not just an unlikely statistical artefact.

### Limitations

Although we have used strict criteria when controlling for multiple comparisons, the reported values for the correlation studies are no bigger than 0.45 corresponding to a weak-to-moderate linear relationship. This could be explained on the basis of a) the subtle functional implications of the frontal aslant connections and b) the S900 dataset consisting of healthy subjects; the strategy of sampling the extremes of the distribution works only as a rough tendency approximation to what could be expected in an actual comparison between a pathological group and a healthy control group. Given that no subject in this study presents language or working memory impairments, the classical criterion of considering r~ = 0.4 as a weak-moderate relationship could be too conservative. Studies using pathological samples should yield stronger results and an improvement in accuracy scores in the classification tasks should also be expected.

Additionally, a machine learning model could be fed with a large number of parameters and this could improve the resulting classification accuracy, but choosing the parameters is a nontrivial task that demands a deeper understanding of the fundamental aspects of the exFAT structure before it can be safely approached.

The language task proposed by Binder *et al*. included in the S900 protocol was designed to elicit strong fMRI activation of the anterior temporal lobe [25], making it unsuitable for assessment of specific language function components. Moreover, language performance scores were strongly biased towards high values (i.e. the test was too easy) thus limiting its discriminative power. Studying the exFAT volumes with more specific and discriminative language tests should help to better understand the exFAT language implications as well as its possible role in the language networks of the brain.

It should be noted that significant results for top vs bottom comparisons are not bilateral, while we failed to reject the null hypothesis that left and right exFAT adjusted volume indices are different. This possibly indicates that for a larger sample size a statistically significant difference between left and right exFAT adjusted volume indices would be found, although its magnitude would be so small that its real life relevance should only become apparent when studying large populations.

### Future work

Further studies should be conducted to assess whether the bilateral exFAT coordinates language and working memory functions: Given that the exFAT is a wide tract that includes the FAT, it could be useful to split the exFAT into its original FAT component and an anterior FAT component in order to elucidate to what extent these separate components have disjoint functional implications. Given the left-lateralized nature of the FAT and the symmetrical nature of the exFAT, the anterior FAT should be expected to be a right-lateralized tract.

## Conclusions

We did not find the exFAT to be lateralized in any of the studied groups. For the extreme groups, we report as significant those results that allow us to reject H_0_ both in a correlation test and in a t-test after applying a very conservative multiple comparison correction, and additionally score at a higher-than-chance level in the holdout set of a machine learning cross-validated classifier model. Namely, we found the left adjusted exFAT volume index to be associated with performance in a general language task and the right adjusted exFAT volume index to be associated with performance in a 2-back working memory task. To our best knowledge, the later observation represents a novel result that has not been previously reported by studying the FAT alone.

Further studies should be conducted to better characterize the microstructural properties of the exFAT, and to understand the specific functional implications of its potential different parts.

In the present study, we presented a methodology based on a high quality open access dataset and free software tools that can be easily replicated and extended to study other white matter tracts. We have shown that sampling extreme subjects from the Human Connectome Project dataset can be a useful strategy for studying the functional implications of brain structure in the healthy human brain.

## Supporting information

S1 Fig Descriptors for global sample. Histograms (1, 2) and scatter plots (3, 4, 5, 6) characterizing the global sample (N = 834) for left (1, 3, 5) and right (2, 4, 6) exFAT adjusted volume indices.
(TIF)

S2 Fig Language extreme groups. Boxplots showing adjusted exFAT volume index distribution in language extreme groups. The triangle indicates the mean value. Whiskers extend 0.5*IQR beyond the first and third quartile lines. Notches indicate confidence interval around the median.
(TIF)

S3 Fig Working memory groups. Boxplots showing adjusted exFAT volume index distribution in working memory extreme groups. The triangle indicates the mean value. Whiskers extend 0.5*IQR beyond the first and third quartile lines. Notches indicate confidence interval around the median.
(TIF)
