# Supplementary figures and images for: When the FAT goes wide: Right extended Frontal Aslant Tract volume predicts performance on working memory tasks in healthy humans
Source: PLoS One. 2018 Aug 1;13(8):e0200786. doi: 10.1371/journal.pone.0200786 (PMC6070228; doi:10.1371/journal.pone.0200786)

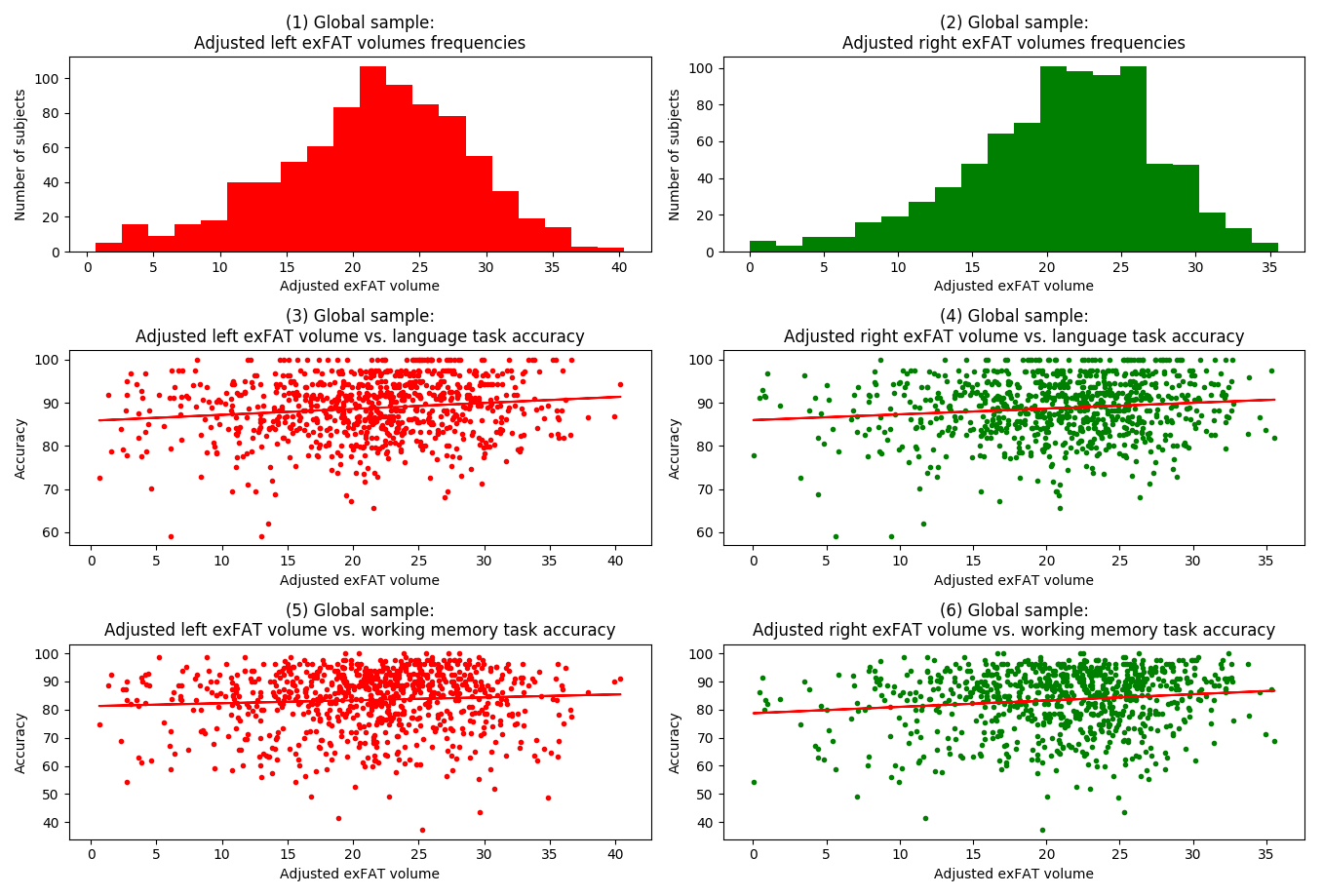

Supplement: S1 Fig — Histograms (1, 2) and scatter plots (3, 4, 5, 6) characterizing the global sample (N = 834) for left (1, 3, 5) and right (2, 4, 6) exFAT adjusted volume indices. (TIF) [file pone.0200786.s001.tif]

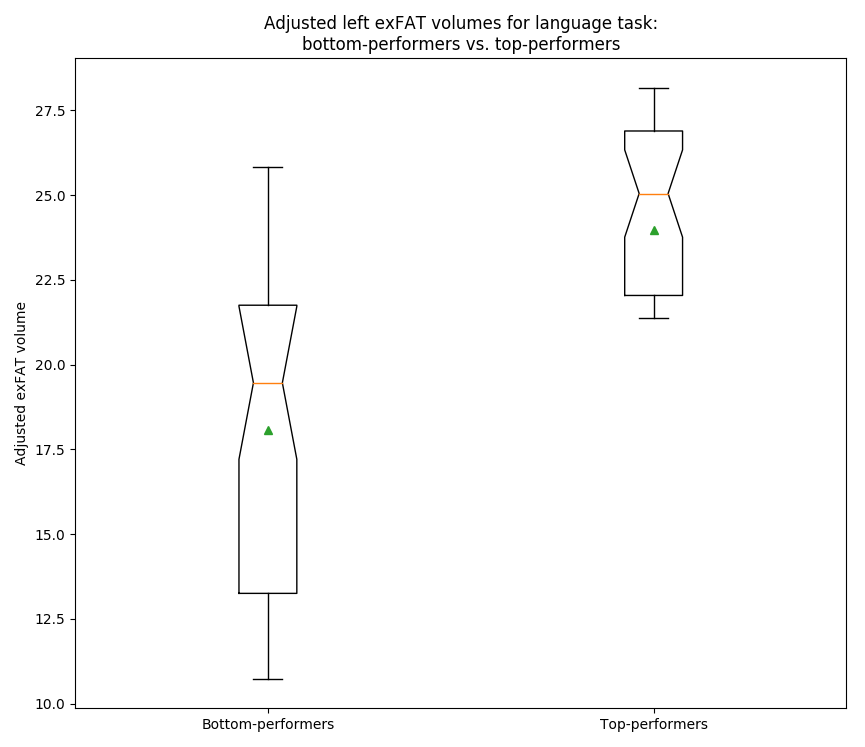

Supplement: S2 Fig — Boxplots showing adjusted exFAT volume index distribution in language extreme groups. The triangle indicates the mean value. Whiskers extend 0.5*IQR beyond the first and third quartile lines. Notches indicate confidence interval around the median. (TIF) [file pone.0200786.s002.tif]

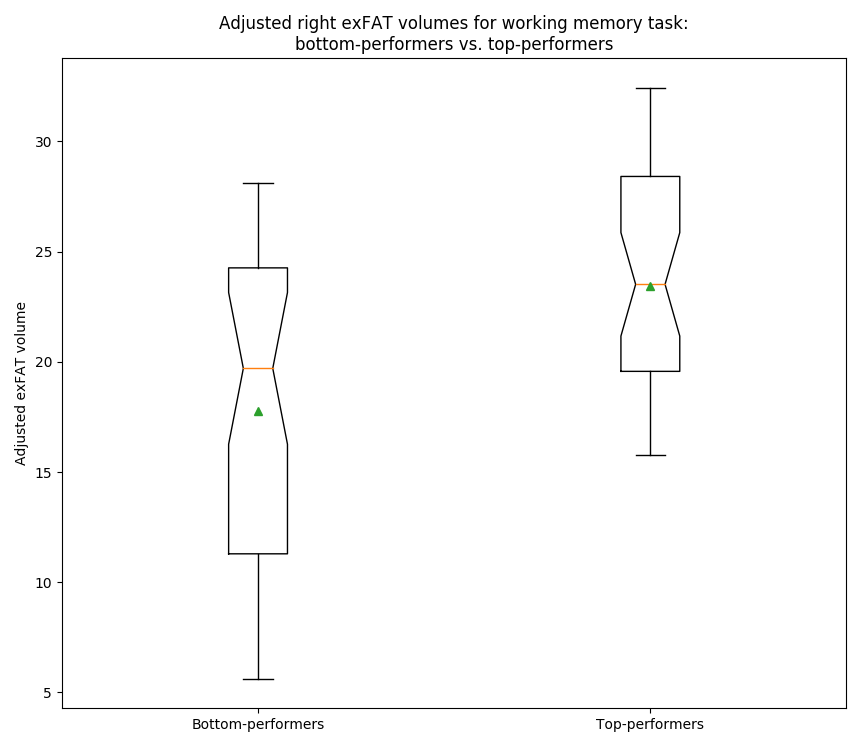

Supplement: S3 Fig — Boxplots showing adjusted exFAT volume index distribution in working memory extreme groups. The triangle indicates the mean value. Whiskers extend 0.5*IQR beyond the first and third quartile lines. Notches indicate confidence interval around the median. (TIF) [file pone.0200786.s003.tif]
